# P-411. Is empiric coverage for methicillin resistant Staphylococcus aureus needed for uncomplicated pediatric bone and joint infections?

**DOI:** 10.1093/ofid/ofaf695.628

**Published:** 2026-01-11

**Authors:** Joel Rose-Kamprath, Alec Wesolowski, Saul I Favela, Marisol Fernandez

**Affiliations:** The University of Texas at Austin Dell Medical School, Austin, TX; Dell Children's Medical Center, Austin, TX; University of Texas at Austin Dell Medical School, Austin, Texas; Dell Children's Medical Center of Central Texas, Dell Medical School at UT Austin, Austin, Texas

## Abstract

**Background:**

*Staphylococcus aureus,* including those that are methicillin resistant (MRSA) is an important etiology of pediatric bone and joint infections (BJI). The prevalence of MRSA associated BJIs is declining nationally. This is likely multifactorial, attributed to improved infection control measures, antibiotic stewardship programs, and changes in virulence factors. Studies have also shown a rising resistance to clindamycin. Our institution’s current BJI guideline recommends empiric clindamycin for MRSA coverage in addition to a beta-lactam antibiotic. Resistance to clindamycin among *Staphylococcus aureus* isolates along with a decline in MRSA infections locally and nationally calls into question the utility of empiric coverage of MRSA for pediatric BJI.

**Figure:**
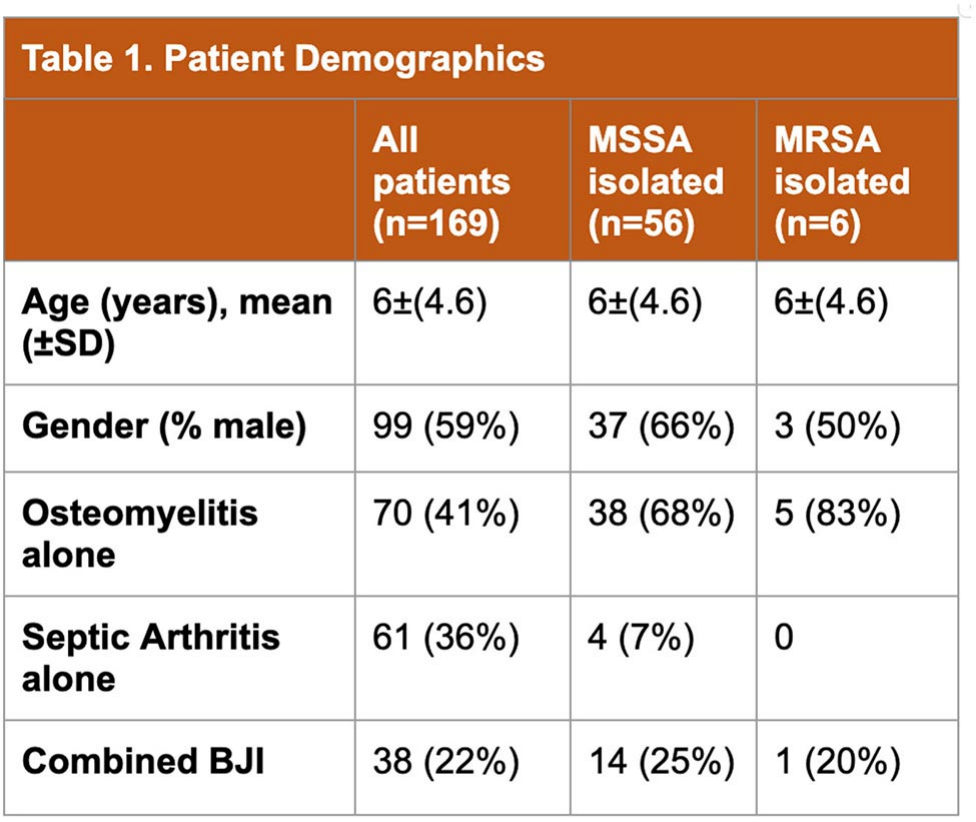

**Figure:**
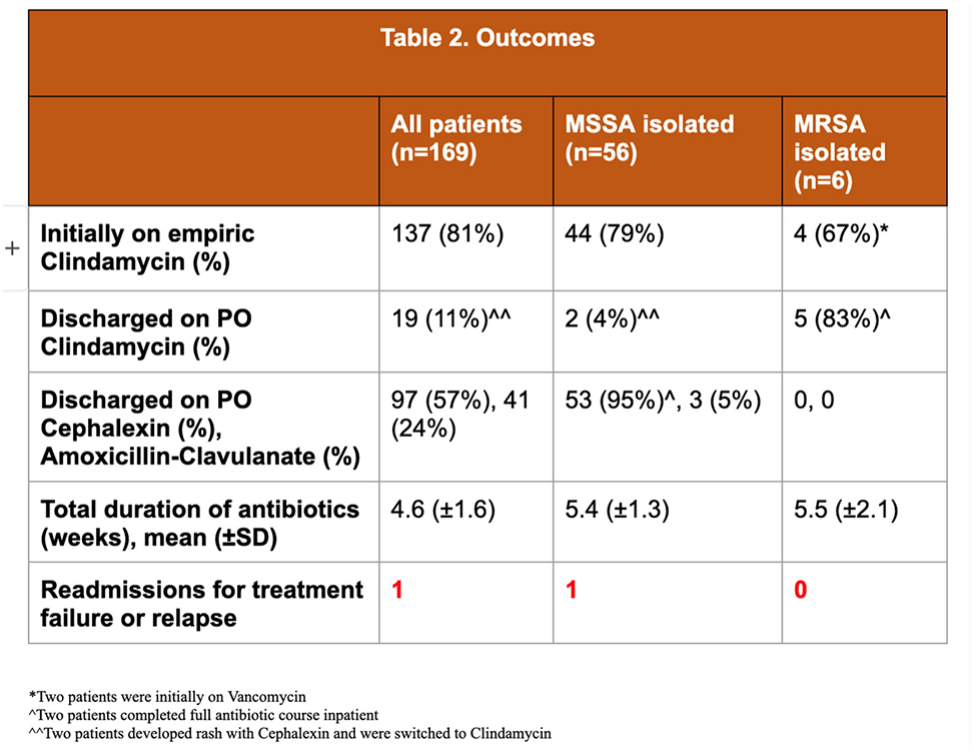

**Methods:**

This single-center, multi-site, retrospective cohort study included pediatric patients admitted between January 2015 and April 2024 in Central Texas who were diagnosed with BJI. Immunocompromised patients, chronic osteomyelitis, post-surgical osteomyelitis and patients older than 18 years were excluded. Patient demographics, organisms isolated, antibiotic susceptibility, initial and final antibiotic regimens were extracted from the charts. Outcomes assessed included 30-day readmission, microbiologic failure, and relapse.

**Figure:**
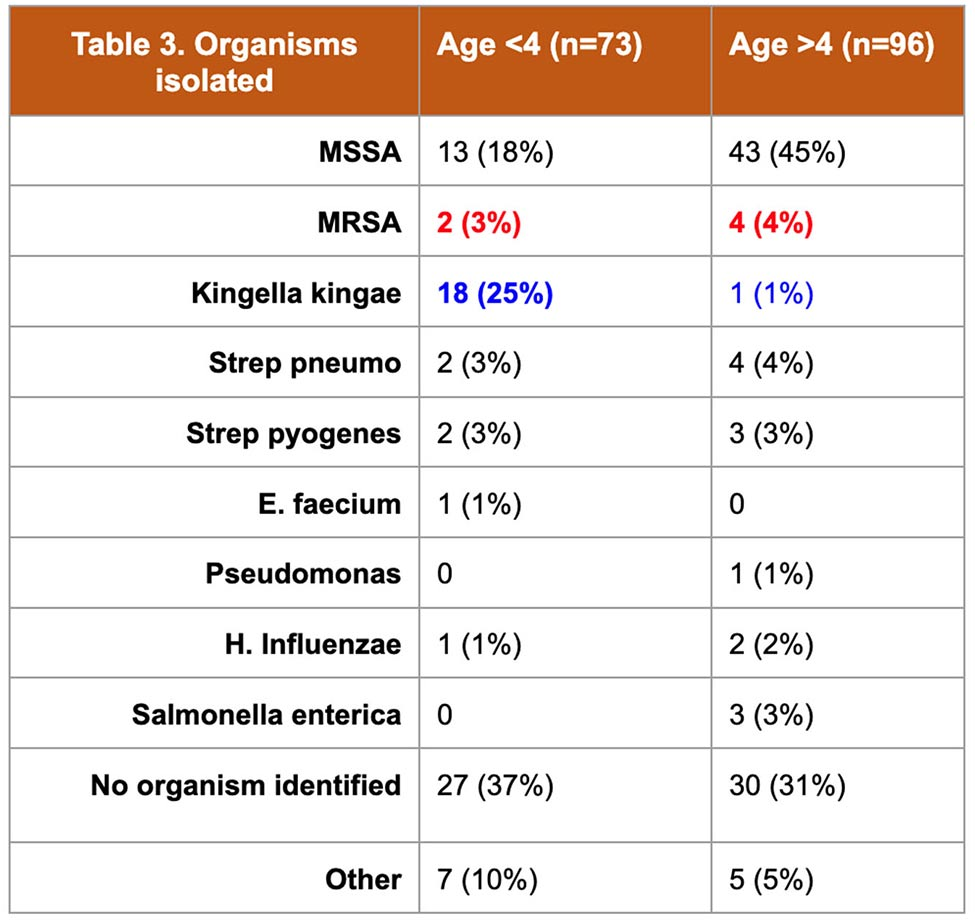

**Results:**

233 patient charts were reviewed and 169 patients were included. A bacterial pathogen was identified in 66% of cases. *S. aureus* was the most common pathogen identified in 62 (37%) cases; 6 (4%) cases were identified as MRSA. 137 (81%) patients received empiric clindamycin. Among the 58 cases without a pathogen identified, 49 (84%) were empirically started on MRSA coverage with clindamycin; 46 (80%) were discharged without antibiotics against MRSA. There was only one readmission for treatment failure or relapse across all patients included.

**Conclusion:**

Our review of 9 years of data on BJIs in Central Texas revealed a low rate of confirmed MRSA (4%). In this sample, 70% of cases were discharged without MRSA coverage, including those without a pathogen identified. Only one case of MSSA required readmission for treatment failure or relapse. The need for empiric MRSA treatment in cases of uncomplicated BJI might not be necessary in areas of low MRSA prevalence.

**Disclosures:**

All Authors: No reported disclosures

